# Ketamine ameliorates activity-based anorexia of adolescent female mice through changes in the prevalence of NR2B-containing NMDA receptors at excitatory synapses that are in opposite directions for of pyramidal neurons versus GABA interneurons In medial prefrontal cortex

**DOI:** 10.21203/rs.3.rs-2514157/v1

**Published:** 2023-01-31

**Authors:** Jennifer Li, Yi-Wen Chen, Chiye Aoki

**Affiliations:** New York University; New York University; New York University

**Keywords:** NR2B, ketamine, prelimbic, activity-based anorexia, post-embed immunogold electron microscopy, synaptic plasticity

## Abstract

A previous study showed that a single sub-anesthetic dose of ketamine (30 mg/kg-KET, IP) has an immediate and long-lasting (>20 days) effect of reducing maladaptive behaviors associated with activity-based anorexia (ABA) among adolescent female mice. This study sought to determine whether synaptic plasticity involving NR2B-containing NMDA receptors (NR2B) at excitatory synapses in the prelimbic region of medial prefrontal cortex (mPFC) contributes to this ameliorative effect. To this end, quantitative electron microscopic analyses of NR2B-subunit immunoreactivity at excitatory synapses of pyramidal neurons (PN) and GABAergic interneurons (GABA-IN) were conducted upon layer 1 of mPFC of the above-described mice that received a single efficacious 30 mg/kg-KET (N=8) versus an inefficacious 3 mg/kg-KET (N=8) dose during the food-restricted day of the first ABA induction (ABA1). Brain tissue was collected after these animals underwent recovery from ABA1, then of recovery from a second ABA induction (ABA2), 22 days after the ketamine injection. For all three parameters used to quantify ABA resilience (increased food consumption, reduced wheel running, body weight gain), 30 mg/kg-KET evoked synaptic plasticity in opposite directions for PN and GABA-IN, with changes at excitatory synapses on GABA-IN dominating the adaptive behaviors more than on PN. The synaptic changes were in directions consistent with changes in the excitatory outflow from mPFC that weaken food consumption-suppression, strengthen wheel running suppression and enhance food consumption. We hypothesize that 30 mg/kg-KET promotes these long-lasting changes in the excitatory outflow from mPFC after acutely blocking the hunger and wheel-access activated synaptic circuits underlying maladaptive behaviors during ABA.

## Introduction

Anorexia nervosa (AN) is characterized by self-starvation, heightened anxiety and excessive exercising (Carrera et al. 2012; Crisp 1967; Kron et al. 1978), culminating in severe body weight loss (Kaye et al. 2009). AN is experienced by approximately 1% of females at least once in their lifetime (Hudson et al. 2007; Keski-Rahkonen et al. 2007; Smink et al. 2014), has the second highest mortality rate among mental illnesses, following opioid addiction (Arcelus et al. 2011), and has an unacceptably high relapse rate of 25–30% (Rieux et al. 2002; Walsh 2013). There is at present no accepted pharmacological treatment for AN (Rieux et al. 2002; Walsh 2013; Barbarich-Marsteller 2012; Crow 2019). Although often comorbid with anxiety (Kaye et al. 2009), anxiolytics have not been efficacious (Steinglass et al. 2014).

Activity-based anorexia (ABA) is an animal model that captures core symptoms of AN, including heightened anxiety, weight loss, excessive exercise, and voluntary food restriction, enabling exploration of the neurobiological bases of AN’s maladaptive behaviors (Aoki 2020; Aoki et al. 2017; Gutierrez 2013; Aoki and Santiago 2022; Scharner and Stengel 2020). We had previously shown a positive correlation between the severity of ABA and the level of NR2B-containing NMDA receptors (NR2B) at excitatory synapses of pyramidal neurons in the hippocampus (Chen et al. 2017a). Prompted by this and another study showing that some patients diagnosed with AN responded positively to an NMDA receptor antagonist, ketamine (Mills et al. 1998), we asked whether ketamine might also reduce ABA’s maladaptive behaviors. Indeed, a single intraperitoneal (IP) injection of ketamine did ameliorate all of the above-listed core symptoms in mice 14–22 days post-injection (Chen et al. 2018), while we also noted individual differences in vulnerability to anorexia-like behaviors and of their responsiveness to the ketamine treatment. Results from this ABA study, as well as the well-recognized efficacy of low doses of ketamine as an antidepressant (Zorumski et al. 2016), prompted clinicians to test the efficacy of ketamine in treating AN. These studies also yielded mixed results, with some but not all patients experiencing clear remission of disordered eating symptoms (Calabrese 2022; Scolnick et al. 2020; Schwartz et al. 2021). Thus, the aim of this study was to determine whether changes in the expression pattern of NR2B at excitatory synapses might be associated with individual differences in vulnerability to ABA and of their responsiveness to ketamine.

Although multiple brain regions are likely to become engaged in ABA’s maladaptive behaviors and responses to ketamine, this study focused on a single brain region: medial prefrontal cortex (mPFC). The rationale for studying the mPFC is that this brain region has been implicated heavily in mental illnesses like AN that stem from anxiety. Compared to controls, the PFC of patients diagnosed with AN exhibit diminished activities at rest and abnormally increased activities when presented with food images (Uher et al. 2004). Using single photon emission computed tomography (SPECT), hypoperfusion of mPFC at normal resting conditions was found for subjects diagnosed with AN, relative to healthy controls (Takano et al. 2001). Moreover, maladaptive food choices by subjects diagnosed with AN is linked to stronger PFC-striatum connectivity (Foerde et al. 2015). In mice, suppression of hyperactivity evoked by food restriction under the ABA model correlates with enhanced synaptic contacts formed by glutamic acid decarboxylase (GAD)-positive GABAergic axon terminals onto cell bodies of pyramidal cells in layer 5 of the mPFC (Chen et al. 2017b; Chen et al. 2016). Chemogenetic activation 3 hours prior to food availability of mPFC pyramidal cells that project to the dorsal medial striatum (mPFC◊DMS) increases food restriction-evoked wheel running, referred to as food-anticipatory activity (FAA) (Gallardo et al. 2014) significantly, while suppression of these cells at the same hour of the day reduces FAA (Santiago et al. 2021). In contrast to the chemogenic modulatability of FAA, chemogenetic modulation of the food consumption-promoting pathway was not possible (Du et al. 2022). Specifically, activation of mPFC pyramidal neurons projecting to the dorsal raphe nucleus (mPFC◊DR) (Jankowski and Sesack 2004) is known to increase feeding behaviors via activation of GABAergic neurons in DR to which these pyramidal neurons project (Nectow et al. 2017) but chemogenetic activation of the mPFC◊DR pathway of ABA animals could not induce feeding: we learned that this was due to the overriding strong inhibition of this pathway by GABAergic neurons in the mPFC (Du et al. 2022).

In this way, data from rodent and human studies point to the mPFC as a key region for an individual’s decision to forage (run) or eat when experiencing starvation. Besides these accumulating evidence regarding the central role of mPFC in AN and ABA’s maladaptive behaviors, a study investigating ketamine’s mechanism as an antidepressant indicates that functional connectivity between the mPFC and ventral hippocampus is necessary for ketamine’s action(Carreno et al. 2016). These findings led us to focus our analysis on the mPFC for detecting changes in synaptic circuitry in response to the experience of ABA following treatments with efficacious versus inefficacious doses of ketamine. Might mPFC show similar positive correlation between the level of NR2B subunits and vulnerability to ABA, as was observed for the hippocampus (Chen et al. 2017a)? This seemed possible, since activities between the hippocampus and mPFC become synchronized when mice are exposed to anxiogenic environments (Adhikari et al. 2010), such as starvation, and mPFC receives inputs from the ventral hippocampus (Adhikari et al. 2011). Another reason for focusing on NR2B was that ketamine’s antidepressant action is occluded by genetic deletion of NR2B-subunits in pyramidal neurons (Miller et al. 2014; Miller et al. 2017) or in GABA-interneurons (GABA-IN) (Gerhard et al. 2020; Gerhard et al. 2016) and NR2B-containing NMDARs in prefrontal cortex have been shown to be important for contextual memory formation (Zhao et al. 2005). Prompted by these findings, we sought to assess whether the redistribution of NR2B at excitatory synapses formed upon pyramidal neurons versus GABA-IN might be evoked by ketamine and be related to ketamine’s efficacy of reducing ABA’s maladaptive behaviors. Finally, we focused our analysis upon layer 1 of mPFC. This choice was based on earlier works indicating that changes in dendritic spine sizes and numbers could be detected within apical tufts of mPFC following ketamine treatment (Deyama et al. 2019; Sarkar and Kabbaj 2016), including the tips of apical dendritic tufts of layer 5 pyramidal neurons near pia (Li et al. 2010)and because layer 1 has the highest density of excitatory synapses, among all layers of cortex (DeFelipe et al. 1999).

## Methods

### Animals

FR started at 1 pm on P41 for ABA1 and on P55 for ABA2. The 2-hour food access period of each FR day started at 7pm and ended at 9pm. Lights were turned on from 7am to 7pm everyday (light phase). Lights were off from 7pm to 7am (dark phase). 3mg/kg or 30mg/kg ketamine were injected at 6pm of ABA1 FR2,1 hr before feeding. FAA (food anticipatory activity) period refers to the hours of 1 pm to 7 pm, when hunger evokes hyperactivity of ABA animals.

### The Activity-based anorexia (ABA) procedure

**Figure 1:**
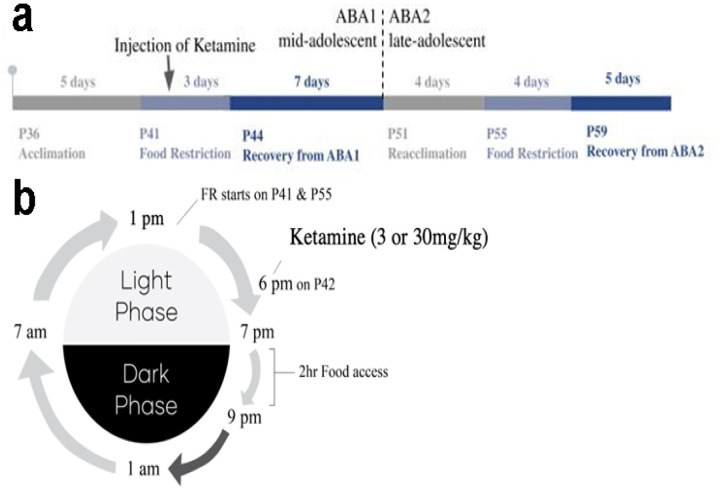
The activity-based anorexia (ABA) animal model **Panel a. The ABA timeline** Acclimation to wheels started on postnatal day 36 (P36) and lasted for 5 days. The first day of food restriction (ABA1 FR1) started on P41 and lasted for 3 days. On ABA1 FR2, which is the second day of ABAI’s food restriction phase (FR), all animals received either 3mg/kg or 30mg/kg of ketamine by intraperitoneal injection. The recovery phase after ABA1 FR started on P44 and lasted for 7 days. Animals were then re-acclimated to wheels for 4 days, starting on P51. Animals were food restricted again (ABA2 FR) for 4 days, starting on P55. The second recovery phase, which followed ABA2 FR, started on P59 and lasted for 5 days. **Panel b. Schematic of the 244ir schedule during FR days** FR started at 1 pm on P41 for ABA1 and on P55 for ABA2. The 2-hour food access period of each FR day started at 7pm and ended at 9pm. Lights were turned on from 7am to 7pm everyday (light phase). Lights were off from 7pm to 7am (dark phase). 3mg/kg or 30mg/kg ketamine were injected at 6pm of ABA1 FR2,1 hr before feeding. FAA (food anticipatory activity) period refers to the hours of 1 pm to 7 pm, when hunger evokes hyperactivity of ABA animals.

Figure 1 shows the timeline of ABA. ABA1 started on postnatal day 36 (P36) for all animals. On this day, group-housed animals began to be singly housed. During P36 to P40, animals underwent acclimation to the addition of a wheel in its cage, and were exposure to wet food (Clear H_2_O brand DietGel 76A in plastic cups, 0.998 kcal/g, 4.7% protein, 17.9% carbohydrates, 1.5% fat, 73.4% moisture), in addition to dry food pellets that they had received before weaning (LabDiet PMI Nutrition Inti, Brentwood, MO’s #5001; 10% fat, 20% protein, 70% carbohydrate, 4.07 gross energy kcal/g, 3.02 metabolizable kcal/g; alternatively LabDiet Rodent Diet 20 EXT (5053); 20% protein, 4.5% crude fat, 6% crude fiber, 7% ash, 12% maximum moisture with 4.07 gross energy kcal/g, equal to 3.07 metabolizable energy kcal/g). This period was used for acquiring baseline wheel running, body weight and food consumption data. These data were collected daily, to the end of ABA2, for monitoring changes in wheel running, body weight and food consumption during different periods of the experiment.

Food restriction (FR) of ABA1 lasted for three days, beginning at 1 pm on P41 (FR1) until 1 pm on P44 (FR3), during which time animals’ food access was limited to 2 hours, from 7 pm to 9 pm. During the hours without food, a water gel cup (Clear H_2_O brand Hydrogels Produce #70-01-5022, 0 kcal/g) was placed where the wet food had been placed during acclimation, and the dry food hopper remained in place but was empty. Thus, food restriction began on P41 by removing both wet and dry food at 1 pm. Food was given at 7 pm, then taken away at 9 pm until 7 pm of the next day. Animals tended to eat less food on the first day of FR (FR1) than on other FR days.

Ketamine administration occurred on P42. On this day, animals were randomly assigned to receive either 3 mg/kg or 30 mg/kg of ketamine. Ketamine was diluted with sterile saline, yielding a volume of 0.2 cc/20 g of body weight) and injected intraperitoneally (i.p.) at 6 pm, one hour before food access that began at 7pm.

Recovery period lasted from P44 to P51. During this period, wheels were taken away and food access returned to being *ad libitum*. Specific days during recovery are referred to in the Results section as R2, R6, etc., referring to the 2nd, 6th day, etc., of recovery, relative to the end of the FR period.

Re-acclimation to the wheel for ABA2 began on P51 and continued for 4 days, until P55. The extent of wheel running and food consumption during the re-acclimation period was used to determine the new baseline for the second FR period of ABA2. The second FR period of ABA2 lasted for four days, from P55 (FR1) to P59 (FR4), during which time relapse of anorexia-like behaviors of excessive exercise, voluntary food restriction and body weight loss were assessed. Finally, starting on P59, all animals underwent a second recovery for 5 days, during which time wheels were removed and food access became *ad libitum*.

The body weights of all animals were measured daily at around 1 pm for non-FR days and at 7pm and 9pm for FR days. The weights of dry and wet food consumption were also measured whenever body weights were measured, then converted to total kilocalories (kCal) consumed per day. Wheel activity was recorded manually whenever body weights were measured. Wheel activity was also recorded at one-minute resolution during all hours of wheel accessibility. For both FR periods, animals were moved to a new cage right after the end of FR. Though all mice were females, the estrous cycle was not monitored because 1. puberty was too immature for a stable estrous cycle, 2. FR disrupts the cycle regardless, and 3. vaginal smears, required to obtain data for estrous cycle, exacerbates anxiety of animals already stressed by FR and considered undesirable perturbations (Chen et al. 2016).

All aspects of animal handling for ABA were performed by Dr. Yi-Wen Chen. Data on body weight, food consumption and wheel counts of these ketamine-injected animals have been published (Chen et al. 2018).

### Elevated Plus Maze

The Elevated Plus Maze (EPM) was used to test for anxiety-like behaviors. This was performed on P49 and P62 during the dark cycle for 10 minutes for each animal. This part of the study using the EPM to measure anxiety-like behaviors of mice was conducted by Dr. Yi-wen Chen and is already published (Chen et al. 2018). These EPM data are not presented in the current study.

### Histological Preparation

On the morning of P63 (± 1 day), all 16 animals were anesthetized with urethane (1000–1500 mg/kg) by IP injection, followed by transcardial perfusion with 50ml of phosphate-buffered saline (PBS; 0.01 M phosphate buffer, 0.9% sodium chloride, pH 7.4) containing Heparin (20 U/ml). This was followed immediately by perfusion with 0.1 M phosphate buffer (pH 7.4) containing 4% paraformaldehyde (PFA, EM Sciences, Hatfield, PA, USA). Glutaraldehyde was not included in the perfusate, to minimize loss of antigenicity of brain proteins. Brains were stored in 4% PFA in 0.1 M phosphate buffer at 4°C until use, 2 years later.

**Figure 2:**
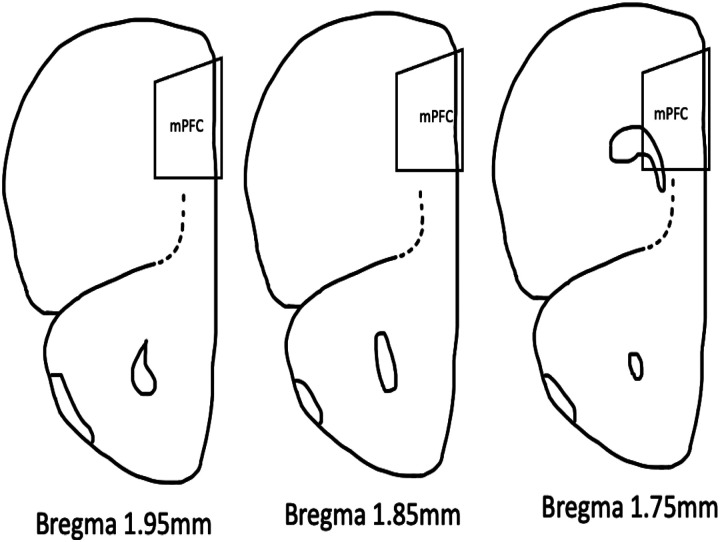
Medial prefrontal cortical regions (mPFC) sampled Schematic representations of Bregma 1,95mm, 1,85mm, and 1,75mm of mice brain. The trapezoid labeled “mPFC’ shows the sampled region.

Brains were sectioned into 2-mm thick coronal slabs using a razor blade, then prepared into coronal sections using a vibrating microtome, set at a thickness of 50 Mm (Leica VT1000M, Leica Microsystems GmbH, Wetzlar, Germany). Sections containing the prelimbic region of mPFC, Bregma 1.7–2.34, centered at Bregma 1.94 (Fig. 2) were collected and stored in multi-wells at 4°C in saline (0.9% NaCI) buffered with 0.01M PB and preserved with 0.05% sodium azide (PBS-azide).

### Immunocytochemistry

The vibratome tissue underwent a pre-embed immunocytochemical procedure to immunolabel fora postsynaptic protein, drebrin A. Immunocytochemical procedures and the electron microscopic data pertaining to drebrin A localization have been published elsewhere (Temizer et al., 2022), and thus, are not presented here. NR2B-subunits of NMDA receptors (NR2B) were detected within these sections by the post-embed immunogold method, after post-fixing vibratome sections in 2% glutaraldehyde in PBS for 30 min at room temperature and the osmium-free procedure for ultrastructural preservation (Aoki et al. 2000; Phend et al. 1995; Kharazia et al. 1996), as detailed below.

### Osmium-free tissue processing

All steps of this process were performed on ice. Vibratome-cut sections were washed in maleate buffer, pH 6.0 (MB) 2 times for 5 minutes each. Sections underwent the following steps to enhance ultrastructural preservation and contrast: immersion in 1 % tannic acid in MB for 40 minutes, followed by two rinses using MB, then 1 % filtered uranyl acetate in MB for 40 minutes, followed by two rinses in MB, then 0.2% iridium tetrabromide in MB for 20 minutes, followed by two rinses using MB, then incubation in 50% ethanol for 5 minutes, 70% ethanol for 3 minutes, then in 1 % para-phenylenediamine hydrochloric acid in fresh 70% ethanol for 15 minutes while protected from light, followed by rinses using 70% ethanol. Tissues were stored overnight at 4° C in 1% filtered uranyl acetate in 70% ethanol, shielded from light, within scintillation vials.

On the following day, sections were prepared for infiltration with the embedding matrix by dehydrating them in progressively higher concentrations of ethanol, up to 100%, followed by immersion in 100% acetone three times for 5 minutes each, 1:1 volume of EMBED812 (EMSciences Catalog #14900) and 100% acetone for 3 hours at room temperature and 1 hour at 50° C. Next, sections were infiltrated in 100% EMBED812 for 4 hours with 1 of the hours at 50° C, then flat-embedded between two sheets of Aclar plastics. Flat-embedded tissues were cured at 60° C for 24 to 36 hours, with weights added on the Aclar sheets to induce flatness of the sections. After re-embedding the sections in Beem capsules, ultrathin sections were prepared using a diamond knife, then collected onto formvar-coated EM grids (400 mesh, nickel thin bars, EMSciences). These ultrathin sections were processed for post-embedded gold (PEG) immunolabeling.

### Primary and secondary antibodies for NR2B immunocytochemistry by the PEG procedure

The primary antibody for the NR2B subunit of NMDAR (GRIN2B gene product) was a rabbit polyclonal antibody (Millipore Corp, Polyclonal Rb Anti-NR2B 06–600, Lot #251403), applied using previously published procedures (Fujisawa and Aoki 2003; Aoki et al. 2005). This antibody identifies the C-terminal amino acids 1437–1456 (KFNGSSNGHVYEKLSSIESDV). Specificity of the antibody was shown based on recognition of a single band from rat brain microsomal preparation of molecular weight ~ 180kD by Western blotting that excludes immunoreactivity to the NR1 subunit or NR2A subunit of NMDARs (Rinaldi et al. 2007). The antigen recognized by this antibody is expressed with a distinct developmental (Corson et al. 2009; Sheng et al. 1994) and subcellular (Aoki et al. 2005; Fujisawa and Aoki 2003) distribution pattern, compared to the NR2A subunit. The secondary antibody for NR2B was goat anti-rabbit IgG conjugated to 10 nm colloidal gold particles, purchased from Electron Microscopy Science (Cat. #25109).

### The post-embed gold procedure (PEG) for detecting NR2B-containing NMDARs (NR2B)

The PEG procedure was used to label NR2B by applying primary and secondary antibodies directly onto ultrathin sections collected on formvar-coated EM grids, as detailed in previous publications from this lab (Aoki et al. 2003; Aoki et al. 2009; Aoki et al. 2000; Aoki et al. 2005; Fujisawa and Aoki 2003; Fujisawa et al. 2006; Chen et al. 2017a). Incubation of ultrathin sections mounted on grids were collected 5–8 hours prior to incubation in the solutions used for the PEG procedure. This timing between the start of the PEG procedure and ultrathin sectioning was important for ensuring that the sections remained adhered securely to formvar coated-grids while also avoiding waning of antigenicity due to air exposure. Fifty-μ I of a 0.1 M Tris-buffered saline with 0.1 % Triton X-100 (TBST)-pH 7.4 droplet was put into each division of a silicon mat. There were 16 ultrathin sections processed in parallel, derived from 16 different animals. All grids were inserted under a droplet of TBST-pH 7.4 with the ultrathin section facing upwards with respect to the droplet. Next, the sections were transferred in the same manner into a droplet of 50 μl of the primary antibody for NR2B diluted in TBST-pH 7.4 at a dilution of 1:100 (10 Mg/ml). Sections were incubated in the primary antibody solution overnight at room temperature within a humidified chamber. On the following day, sections were rinsed three times at 10-minute intervals with 50 μl of TBST-pH 7.4 droplets in the same manner, followed by incubation in a droplet of TBST-pH 8.4. Then the tissues were incubated in the secondary antibody conjugated to 10 nm colloidal gold particles, diluted in TBST-pH 8.4 (1:40). After an hour of incubation at room temperature within a humidified chamber, sections were rinsed with TBST-pH 8.4 three times and 10 minutes each, and then in deionized water three times at 10-minute intervals. Tissues were fixed in 1 % glutaraldehyde diluted in deionized water for 10 minutes, rinsed in deionized water, and air-dried.

Ultrathin sections designated to serve as immunocytochemical controls were collected on formvar-coated grids, strictly in parallel with those sections described above and were processed for PEG labeling in parallel, except that the step for incubating with the primary antibody was substituted by a step of incubating in TBST-pH 7.4 that lacked the primary antibody.

### Counterstaining

Counterstaining was done in order to increase the contrast of tissues, to facilitate interpretation of the ultrastructure. Lead citrate was prepared as described by Reynolds (Reynolds 1963). A few droplets of lead citrate, using a syringe and filter, were put on a piece of dental wax plate within a petri dish lined with sodium hydroxide pellets. Each EM grid with a tissue on it was immersed in a new droplet of lead citrate for 20 seconds. Immediately after, the tissues on the grid were washed in four beakers of deionized water, then dried on a piece of filter paper. Grids were air-dried for a minimum of 2 hours before viewing under the EM or placed in grid storage containers.

### Electron Microscopy

Layer 1 of the prelimbic area of mPFC was identified by the absence of pyramidal cells and presence of pia mater. All images were magnified to 40,000x, within 5 Mm from cortical surface, then digitally captured using a Hamamatsu CCD camera, attached to a JEOL 1200XL electron microscope, developed by AMT (Boston, MA). Images were taken and analyzed systematically and strictly in the order of encounter, without knowledge of the animal’s behavioral characteristics. Images of 200 synapses (± 5%) were captured from each animal’s mPFC. Excitatory synapses were identified based on the presence of thick postsynaptic densities (PSD) and clusters of vesicles approximately 50 nm in diameter in the opposing presynaptic axon terminal (Peters et al. 1991). The postsynaptic element was determined to be spines, based on the absence of mitochondria and microtubules (Peters et al. 1991). Excitatory synapses on dendritic shafts of GABAergic interneurons were identified by the presence of mitochondria and microtubules and the existence of thick PSDs opposite to axon terminals filled with vesicles (White and Keller 1989). Inhibitory synapses were identified by the cluster of vesicles within the presynaptic terminal and absence of thick PSDs. Images were analyzed and annotated on ImageJ bundled with Java 1,8.0_172. Figures containing electron micrographs were prepared using Adobe Photoshop 2023 version.

### Quantification of the ultrastructural localization of NR2B

**Figure 3:**
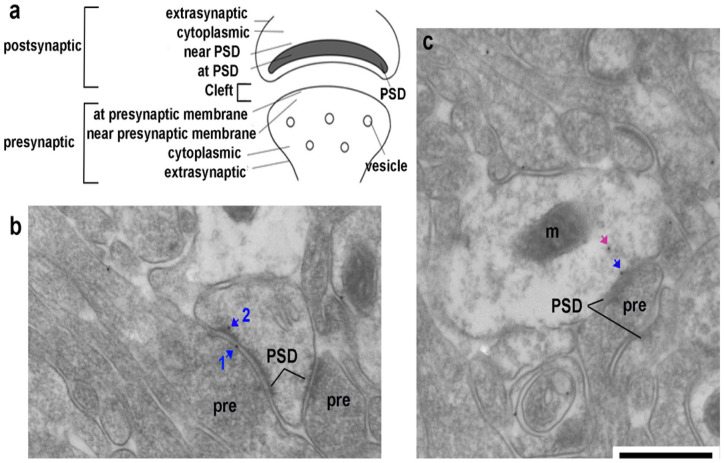
Categorization of NR2B immunolabeling at and near excitatory synapses **Panel a.** Schematic representation of an excitatory synapse, showing the presynaptic and postsynaptic sides. The location of NR2B immunoreactivity was categorized as being at cleft, at extrasynaptic membrane on the pre- or postsynaptic side, cytoplasmic on the pre- or postsynaptic side, near the postsynaptic density (PSD), at the PSD, near the presynaptic membrane or at the presynaptic membrane. **Panel b** shows an example of an axo-spinous excitatory synapse belonging to a pyramidal neuron, evident by the prominent PSD and absence of microtubules or mitochondrial profile in the spine head. PEG labeling reflective of NR2B immunoreactivity is located at the cleft (gold particle 1, blue arrow) and at the PSD (gold particle 2, blue arrow), pre = presynaptic. **Panel c** shows an example of an excitatory synapse on a dendritic shaft, based on the presence of a mitochondrial profile on the postsynaptic side (m) and PSD. One PEG labeling is at the PSD (blue arrow). Another is cytoplasmic (pink arrow). This dendritic shaft is presumed to belong to a GABA-IN (White & Keller, A. 1989). NR2B immunolabels appearing at locations highlighted with blue arrowheads are presumed to be functional since they were on the plasma membrane, while those at locations highlighted with pink arrowheads are presumed to be of reserve or recycling pool of NR2B. Calibration bar = 500 nm and applies to both panels b and c.

NR2B locations relative to excitatory synapses were quantified using digitized electron micrographs, off-line, guided by the ultrastructural categorization of subcellular locations as shown in Fig. 3a. Excitatory synapses occurred at heads of dendritic spines, presumed to belong to pyramidal neurons. Excitatory synapses occurring along dendritic shafts were presumed to belong to GABAergic interneurons (GABA-IN) (White and Keller 1989).

Figures 3b and 3c show examples of PEG immunolabels for NR2B. PEG labels were identified by defined circular black dots that were 10 nm in size, as expected. In both panels of Fig. 3 and for every excitatory synapse identified in this study, PSD was detectable as a thick dark band along the intracellular surface of the plasma membrane opposed to the plasma membrane of vesicle-containing axon terminals.

NR2B immunolabel appeared *on the extrasynapticplasma membrane of* both pre- and postsynaptic sides, *in the cytoplasm* of both pre- and postsynaptic sides, near(~ 10 nm from) the lipid bilayer of plasma membrane and *at the synaptic plasma membrane* (< 10 nm from the lipid bilayer of the synaptic plasma membrane) on the presynaptic sides, near(~ 10 nm) *and over*(< 10 nm) the PSD of the postsynaptic side. They also appeared *in the synaptic cleft*, i.e., within the extracellular space in between the presynaptic and postsynaptic membranes of a synapse. This extracellular cleft location has been reported earlier for glutamate receptors detected by the PEG procedure, presumed to reflect the displacement of colloidal gold from the antigenic site due to the bridging of the primary and secondary antibody IgG molecules (ca. 15 nm in length, each) and of colloidal gold that are 10 nm in diameter (Valtschanoff & Weinberg, 2001). PEG particles associated with mitochondria were regarded as nonspecific labeling and excluded from counting in the cytoplasm.

NR2B levels of each animal were quantified in two ways: (1) as the proportion of all synapses encountered that were immunolabeled by at least one PEG particle at each of the subcellular locations; (2) as the summed total counts of PEG particles at each of the subcellular locations, per the total number of synapses encountered. Excitatory synapses were considered immunolabeled if they were associated with one or more PEG particle. Using this criterion, analysis of over 200 excitatory synapses from control ultrasections that were incubated with TBST lacking the primary antibody resulted in zero immunolabeling, since none were associated with PEG particles.

### Statistical Analysis

Data was processed using an original algorithm created on Python Language Reference, version 3.6.9, on Google Colab. Pearson’s correlation analysis was performed on Graphpad Prism Version 7.01 to identify correlations between body weight, food consumption, wheel counts and prevalence of NR2B at excitatory synapses. Statistical significance was determined by p < 0.05 and R < −0.7 or R > 0.7, and found using another original program created on Python Language Reference, version 3.6.9, on Google Colab. Two sample mean t-tests were performed to analyze group behavioral differences, where p < 0.05 was considered as statistically significant, using GraphPad Prism Version 7.01. Graphs were prepared using GraphPad Prism.

## Results

The behavioral data of the animals whose brains were anatomically analyzed in this study have already been published (Chen et al. 2018). These behavioral data will be presented here as correlations to the anatomical findings from the current study. The current study examined correlations between three ante mortem values - wheel running, food consumption and body weight - and the levels and locations of NR2B in relation to excitatory synapses in layer 1 of the prelimbic area of mPFC. NR2B was labeled by the PEG method.

### Areal Density of Excitatory Synapses

**Figure 4:**
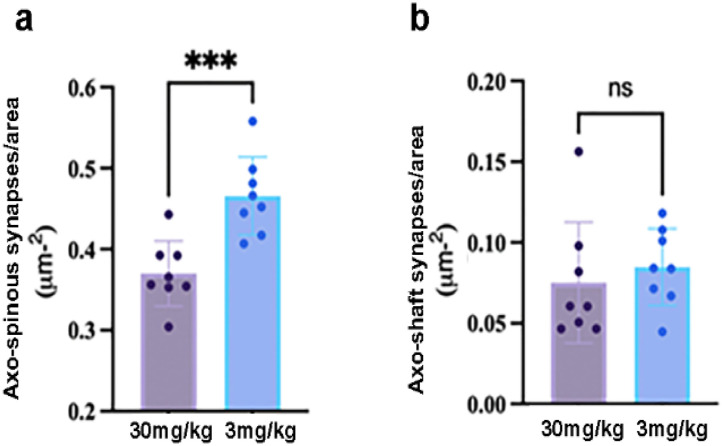
Areal density of excitatory synapses in layer 1, centered in layer 1 a **Panel a.** The 30mg/kg cohort showed significantly less axo-spinous excitatory synapses per area, presumably of pyramidal neurons. **Panel b.** There was no difference across the 30mg/kg versus 3mg/kg cohorts in the areal density of excitatory synapses on dendritic shafts, presumably of GABA-IN.

We compared the areal density of excitatory synapses belonging to pyramidal neurons and GABA-IN in layer 1 (mostly layer 1 a, within 50 Mm from pial surface) of the mPFC. Layer 1 of the 30 mg/kg cohort exhibited a significantly lower density of axo-spinous excitatory synapses (presumably of pyramidal neurons (White and Keller 1989)) than the 3 mg/kg cohort (Fig. 4a, mean ± SEM: 0.3700 ± 0.0143 for the 30 mg/kg cohort, 0.4656 ± 0.0170 for the 3 mg/kg cohort, p = 0.0007). The probability of encounter with randomly positioned objects would be lower for smaller objects (Sterio 1984). However, there was no significant difference in the average spine area between the two cohorts, calculated by averaging 50 spine areas of each animal (mean ± SEM in units of synapses per Mm^2^: 226.2 ± 58.0 for the 30 mg/kg cohort; 341.1 ± 86.3, for the 3 mg/kg cohort, p = 0.484). This suggests that the lower areal density of axo-spinous excitatory synapses seen in the 30 mg/kg cohort did not result from decrease of spine sizes.

The number of axo-shaft excitatory synapses (presumably of GABA-IN (White and Keller 1989)) did not differ significantly between the two cohorts (Fig. 4B, mean ± SEM in units of synapses per Mm^2^: 0.0751 ± 0.0132 for the 30 mg/kg cohort; 0.0847 ± 0.00845, for the 3 mg/kg cohort, p = 0.5503).

### The Proportion of Excitatory Synapses Immunolabeled for NR2B Postsynaptically

Control ultrathin sections that were incubated in buffer lacking the primary antibody showed no PEG labeling at or near any of the 200 excitatory synapses that were sampled. This observation indicated that excitatory synapses with one or more PEG particles could be considered immunolabeled.

**Figure 5:**
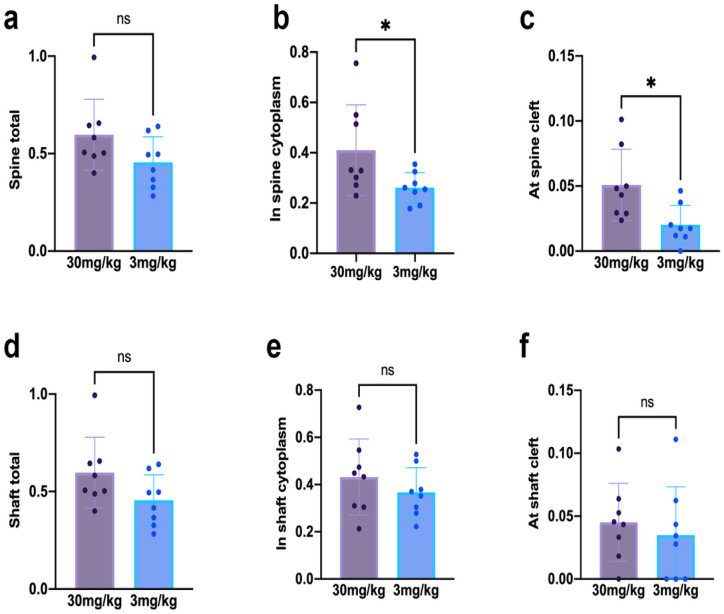
The proportion of excitatory synapses with NR2B immunoreactivity The 30mg/kg cohort showed a significantly higher proportion of postsynaptic NR2B in spine cytoplasm **(panel b)** and synaptic clefts **(panel c)** of pyramidal neurons’ excitatory synapses, compared to the 3 mg/kg cohort but no difference in the proportion labeled, when combining all possible locations at axo-spinous synapses postsynaptically **(panel a).** In contrast, there were no significant differences across the cohorts in the proportion of postsynaptic NR2B anywhere postsynaptically **(panel d),** in the cytoplasm **(panel e)** or in synaptic clefts **(panel f)** near excitatory synapses of dendritic shafts of GABA-IN between the two cohorts.

The proportion of excitatory synapses immunolabeled by NR2B was not different across cohorts, based on tallies of PEG particles across all synaptic locations - namely at and near the PSD, on extrasynaptic portions of the plasma membrane, or in the synaptic cleft or cytoplasm of spines forming excitatory synapses (p = 0.0956; mean ± SEM of 0.5968 ± 0.0643 for the 30 mg/kg cohort; 0.4553 ± 0.04619 for the 3 mg/kg cohort) or at excitatory synapses on dendritic shafts of GABA-IN (p = 0.0643; 0.5968 ± 0.0643 for the 30 mg/kg cohort; 0.4553 ± 0.04619 for the 3 mg/kg cohort). Also, no group difference was found, when the PEG particles were tallied for membranous locations (at the extrasynaptic membrane, at or near the PSD). However, closer inspection revealed that the 30 mg/kg cohort exhibited a significantly higher proportion of spines immunolabeled for NR2B at the cleft and in the cytoplasm of spines (Fig. 5b, mean ± SEM at spines’ excitatory synaptic clefts: 0.0485 ± 0.0088 for the 30 mg/kg cohort, 0.0194 ± 0.0047 for the 3 mg/kg cohort, p = 0.0114; Fig. 5c, mean ± SEM of spine heads with PEG particles in cytoplasm: 0.254 ± 0.028 for the 30 mg/kg cohort, 0.186 ± 0.012 for the 3 mg/kg cohort, p = 0.0410).

At excitatory synapses of dendritic shafts of GABA-IN, no differences in the proportion immunolabeled for postsynaptic NR2B were found between the two cohorts at synaptic clefts (Fig. 5e, mean ± SEM: 0.0450 ± 0.0109 for the 30 mg/kg cohort, 0.0349 ± 0.0136 for the 3 mg/kg cohort, p = 0.571), in the cytoplasm near excitatory synapses (Fig. 5f, mean ± SEM: 0.432 ± 0.0569 for the 30 mg/kg cohort, 0.367 ± 0.0370 for the 3 mg/kg cohort, p = 0.353) or on the plasma membrane of the pre- or postsynaptic sides (not shown).

The level, rather than the proportion, of NR2B immunolabeled per excitatory synapse on dendritic spines and dendritic shafts yielded similar results. Specifically, the level of NR2B labeled at synapses of dendritic spines were significantly greater for the 30 mg/kg cohort, compared to the 3 mg/kg cohort at synaptic clefts (mean ± SEM: 0.0508 ± 0.0097 for the 30 mg/kg cohort, 0.0202 ± 0.0053 for the 3 mg/kg cohort, p = 0.0149) and in the cytoplasm (mean ± SEM: 0.410 ± 0.064 for the 30 mg/kg cohort, 0.261 ± 0.021 for the 3 mg/kg cohort, p = 0.0425) but not at the postsynaptic plasma membranes. There were no differences in the level of NR2B levels between the two cohorts at clefts of excitatory synapses on dendritic shafts (mean ± SEM: 0.0507 ± 0.012 for the 30 mg/kg cohort, 0.0395 ± 0.018 for the 3 mg/kg cohort, p = 0.610), in cytoplasm (0.916 ± 0.22 for the 30 mg/kg cohort, 0.534 ± 0.065 for the 3 mg/kg cohort, p = 0.122) or elsewhere, either.

### Correlational Analyses of the Proportion of Excitatory Synapses Immunolabeled for NR2B with Food Consumption

Within the mPFC, the activation of pyramidal neurons projecting to dorsal raphe (DR) have been shown to promote food consumption of animals undergoing ABA, while GABAergic inhibition of this population of mPFC pyramidal neurons has been shown to underlie suppression of food consumption (Du et al. 2022). This finding prompted us to analyze the possible link between ketamine-mediated increase in food consumption of animals that had experienced ABA and the excitatory synapses formed on pyramidal neurons and GABA-IN of the mPFC. Compared to the control group with vehicle injection (not included in this anatomical analysis), the two ketamine cohorts ate significantly more during the food-restricted period of ABA1, FR2, corresponding to the day that ketamine was injected. Additionally, the 30 mg/kg cohort, but not the 3 mg/kg cohort also ate more on the day following the single ketamine injection, namely FR3 of ABA1. During ABA2,14 days after ketamine injection, only the 30 mg/kg ketamine cohort ate significantly more than the control group during the food-restricted period of ABA2, spanning FR2 through FR4 (Chen et al. 2018). Prompted by these differences in food consumption behaviors across the two ketamine cohorts, correlation analyses between food consumption and the proportion of dendritic spines and shafts immunolabeled for postsynaptic NR2B at excitatory synapses during ABA1 FR, ABA2 FR, and recovery phases were conducted to determine whether individual differences in NR2B expression at excitatory synapses could have contributed to individual differences in the responsiveness to ketamine in terms of food consumption.

#### Correlations at axo-spinous excitatory synapses of pyramidal neurons.

**Figure 6:**
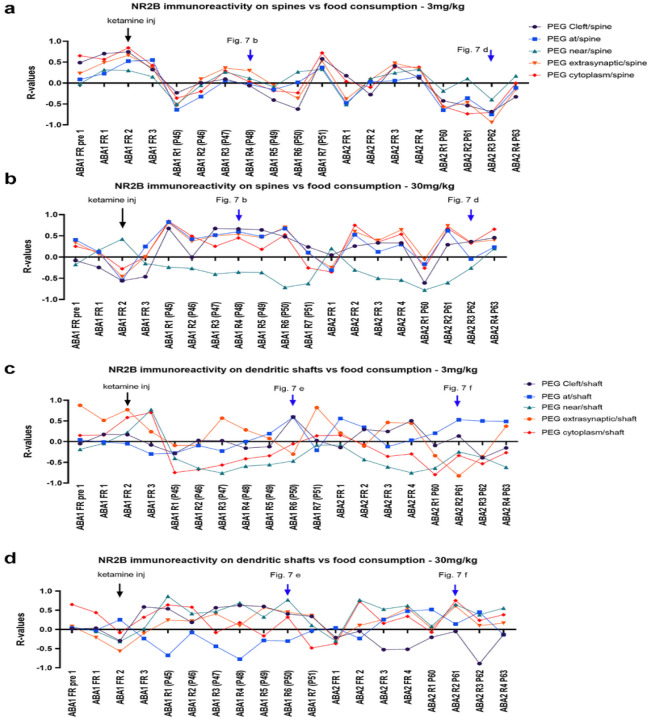
R-values of Pearson correlation analyses throughout the course of ABA1 and ABA2 between food consumption and proportions of NR2B on the postsynaptic side of excitatory synapses **Panels a and b:** axo-spinous synapses on pyramidal neurons. 3mg/kg cohort’s R values are shown panel a, while the 30mg/kg cohort’s R values are shown in panel b. **Panels c and d:** Axo-shaft synapses on GABA-IN. 3mg/kg cohort’s R-values are shown in panel c, while the 30mg/kg cohort’s R-values are shown in panel d.

**Figure 7:**
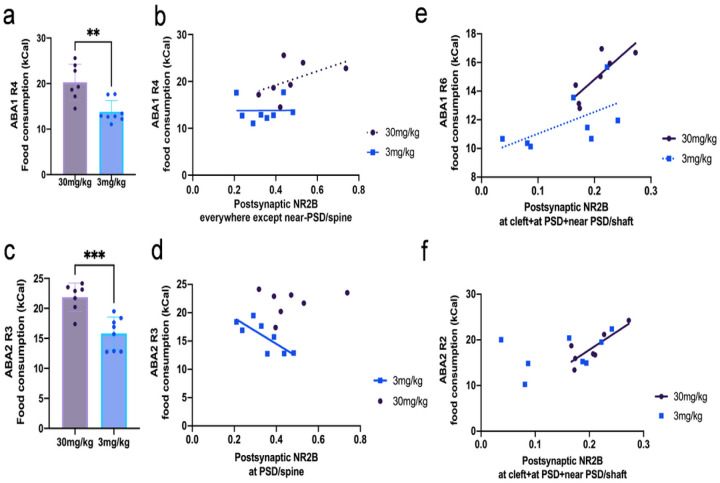
The cohort receiving 30 mg/kg exhibited higher food consumption during recovery from ABA lost NR2B at excitatory synapses of pyramidal neurons and gained NR2B at excitatory synapses of GABA-IN **Panels a and c:** The 30mg/kg cohort exhibited significantly higher food consumption than the 3mg/kg cohort on ABA1 R4 and ABA2 R3. **Panel b:** On ABA1 R4, there was a trend for a positive correlation between food consumption and the proportion of postsynaptic NR2B at all locations except near PSD in dendritic spines for the 30 mg/kg cohort, and no correlation for the 3mg/kg cohort. **Panel d:** On ABA2 R3, there was a significant negative correlation between food consumption and the proportion of postsynaptic NR2B at all locations except near PSD in dendritic spines of the 3 mg/kg cohort and no longer any correlation for the 30 mg/kg cohort. **Panels e and f:** There was a significant positive correlation between food consumption and the proportion of postsynaptic NR2B at cleft, at PSD, and near PSD of excitatory synapses on GABA-IN shafts of the 30 mg/kg cohort on ABA1 R6 and ABA2 R2. In contrast, the 3 mg/kg cohort showed only a positive trend on ABA1 R6 and no correlation on ABA2 R2.

Fig. 6 shows the R values of correlations between the proportion of excitatory synapses with postsynaptic NR2B levels and food consumption recorded for each of the days spanning ABA1 and ABA2. There were clear differences in the patterns between the two ketamine cohorts (Fig. 6a and b). The two time points that showed strong divergence between the cohorts were ABA2 R3 and ABA1 R4, which were examined more closely (Fig. 7).

Closer inspection of ABA2 R3 revealed that the 30 mg/kg cohort showed significantly higher food consumption than the 3 mg/kg cohort (mean ± SEM in units of kcal: 21.9 ± 0.894 for the 30 mg/kg cohort, 15.8 ± 0.962 for the 3 mg/kg cohort, p = 0.0005 by unpaired t-test, Fig. 7c). The 30 mg/kg cohort showed no correlation between NR2B immunoreactivity at dendritic spines and food consumption on any single day of recovery or average consumption across the days of recovery. In sharp contrast to the 30 mg/kg cohort, the 3 mg/kg cohort showed a significantly negative correlation between food consumption and the proportion of spines immunolabeled for postsynaptic NR2B at all locations except for near PSD (Pearson’s R = 0.2117, p = 0.6486 for the 30 mg/kg cohort; Pearson’s R=-0.7892, p = 0.020 for the 3 mg/kg cohort, Fig. 7d). The negative correlation indicates that those individuals consuming the most were those that expressed NR2B minimally at excitatory synapses on pyramidal neurons. These findings indicate, together, that enhanced excitability of pyramidal neurons dictated suppression of food consumption of the 3 mg/kg cohort. Conversely, the 30 mg/kg cohort was likely to have enhanced food consumption through weakening of food-suppressing excitatory pathway(s) that remained active in the brains of the 3 mg/kg cohort (discussion expanded with accompanying figure in the Discussion section).

By comparison, for both cohorts, correlations between food consumption and NR2B-immunoreactivity at dendritic spines were much weaker during recovery from ABA1 (Fig. 7b). For example, on ABA1 R4, when the 30 mg/kg cohort had significantly higher food consumption than the 3 mg/kg cohort (mean ± SEM in units of kcal: 20.3 ± 1.50 for the 30 mg/kg cohort, 13.81 ± 0.87 or the 3 mg/kg cohort, p = 0.002 by unpaired t-test, Fig. 7a), the 30 mg/kg showed only a weakly positive trend between food consumption and the proportion of spines immunolabeled for postsynaptic NR2B at all locations excluding the non-membranous near-PSD category, while the 3mg/kg cohort showed no correlation or trend (Pearson’s R = 0.5046, p = 0.2483 for the 30 mg/kg cohort, Pearson’s R = 0.0070, p = 0.9868 for the 3 mg/kg cohort, Fig. 7b). Similarly, on ABA1 R6, the 30 mg/kg cohort had significantly higher food consumption than the 3 mg/kg cohort (mean difference between cohorts ± SEM in units of kcal of 3.18 ± 0.928, p = 0.0045 on ABA1 R6 unpaired t-test), but the 30 mg/kg cohort showed only a weakly positive trend while the 3 mg/kg cohort exhibited no correlation or trend (on ABA1 R6 Pearson’s R = 0.4756, p = 0.2807 for the 30 mg/kg cohort, and Pearson’s R = 0.1146, p = 0.7870 for the 3 mg/kg cohort for all locations excluding the non-membranous near-PSD category). The lack of correlations during ABA1 indicates that pyramidal neurons either became disengaged in the regulation of food consumption and/or did not contribute in any uniform way towards the enhanced food consumption elicited by 30 mg/kg of ketamine.

#### Correlations at axo-shaft excitatory synapses of GABA-IN.

Unlike the correlations found in pyramidal neurons, the correlations at excitatory synapses of GABA-IN appeared during recovery days after both ABA1 (Fig. 7e) and ABA2 (Fig. 7f) and only for the 30 mg/kg cohort. Specifically, on ABA1 R6 and ABA2 R2, a significantly positive correlation was found in the 30mg/kg cohort between food consumption and the proportion dendritic shafts immunolabeled for postsynaptic NR2B at the cleft, at the PSD, and near the PSD (Pearson’s R = 0.8058, p = 0.0288 for the 30 mg/kg cohort on ABA1 R6, Fig. 7e; Pearson’s R = 0.8231, p = 0.0229 for the 30 mg/kg cohort on ABA2 R2, Fig. 7f), indicating that those individuals with the greatest capacity to consume food expressed NR2B at excitatory synapses on GABA-IN most robustly, presumably suppressing the mPFC’s excitatory outflows that mediate suppression of food consumption. In contrast, for the 3 mg/kg cohort, only a weakly positive correlation trend was found on ABA1 R6 (Pearson’s R = 0.5888, p = 0.1246 for the 3 mg/kg cohort, Fig. 7e), and no trend was found on ABA2 R2 (Pearson’s R = 0.3901, p = 0.3394 for the 3 mg/kg cohort, Fig. 7f). This lack of trend for the 3 mg/kg cohort supports the interpretation that the inefficacious dose could not trigger the increased expression of NR2B at excitatory synapses of GABA-IN for suppressing the food consumption-suppressive excitatory outflow. The appearance of correlations for the NR2B-immunoreactivity during ABA1 for axo-shaft excitatory synapses of GABA-IN and not for the excitatory synapses of pyramidal neurons’ dendritic spines indicates that excitatory synapses on GABA-IN responded more quickly to the 30 mg/kg ketamine injection than the pyramidal neurons.

All data reported above were analyzed based on values of the proportion of dendritic spines or shafts immunolabeled for NR2B at excitatory synapses. Correlation analyses performed by counting every single NR2B immunolabel and normalizing the sum by the number of axo-spinous or axo-shaft excitatory synapses (i.e., NR2B levels) also yielded significance for the same days of food consumption.

Together, these data indicate that the long-lasting gain of ABA resilience by the 30 mg/kg dose, measured as an increase of food consumption more than 20 days after the single injection, correlated with increases of NR2B-immunoreactivity at excitatory synapses of GABA-IN and with the loss of correlation of NR2B-immunoreactivity at axo-spinous synapses. Conversely, we hypothesize that the lack of an ameliorative effect of the 3 mg/kg dose during ABA2 is likely to have been due, in part, to the failure of the mPFC of these animals to de-couple the mPFC synaptic circuitry linking axo-spinous synapses with suppression of food consumption and/or enhance NR2B expression at excitatory synapses on GABA-IN that suppress the excitatory outflow from mPFC that suppress food consumption (discussion is expanded in the Discussion section, with accompanying figure).

### Correlations Between Wheel Activity and the Proportion of Excitatory Synapses Immunolabeled for NR2B Postsynaptically

Earlier work from this lab showed that the mPFC pyramidal neurons projecting to dorsal medial striatum positively modulate wheel running of animals experiencing ABA while inhibition by GABAergic interneurons in mPFC suppresses wheel running (Santiago et al. 2021). We also reported earlier that both ketamine cohorts ran significantly less than the control group during ABA2 but not during ABA1 (Chen et al. 2018). These observations prompted us to assess correlations between wheel activity during ABA2 and the proportion of dendritic spines and shafts immunolabeled for postsynaptic NR2B at excitatory synapses in the mPFC of the two ketamine cohorts.

#### Correlations at axo-spinous excitatory synapses of pyramidal neurons.

**Figure 8:**
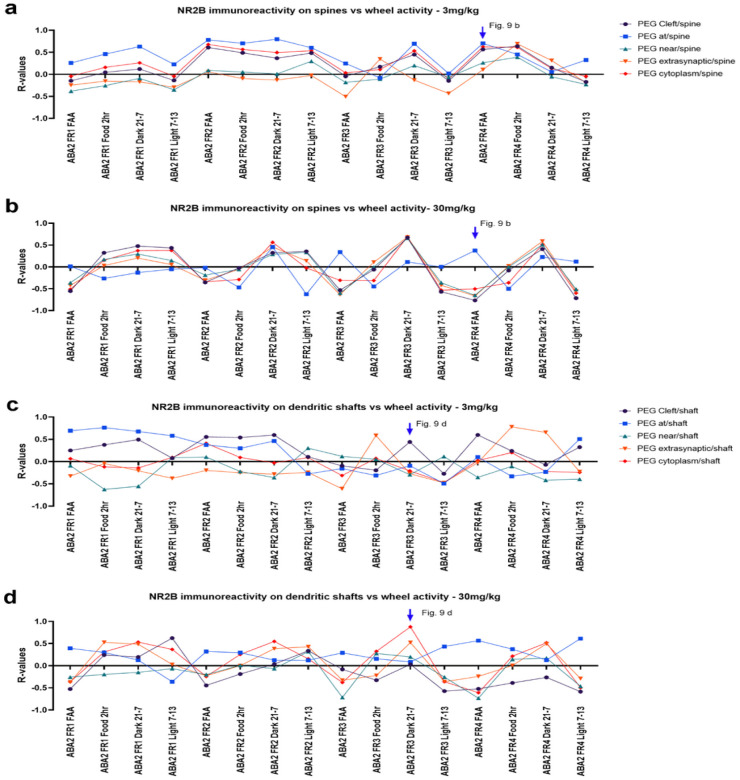
R-values of Pearson correlation analyses throughout the course of ABA1 and ABA2 between wheel activity and proportions of NR2B at excitatory synapses **Panels a and b:** axo-spinous synapses on pyramidal neurons. 3mg/kg cohort’s R values shown in panel 30mg/kg cohort’s shown in panel b. **Panels c and d:** Axo-shaft synapses on GABA-IN. 3mg/kg cohort’s R-values shown in panel c, 30mg/kg cohort’s R-values shown in panel d.

**Figure 9:**
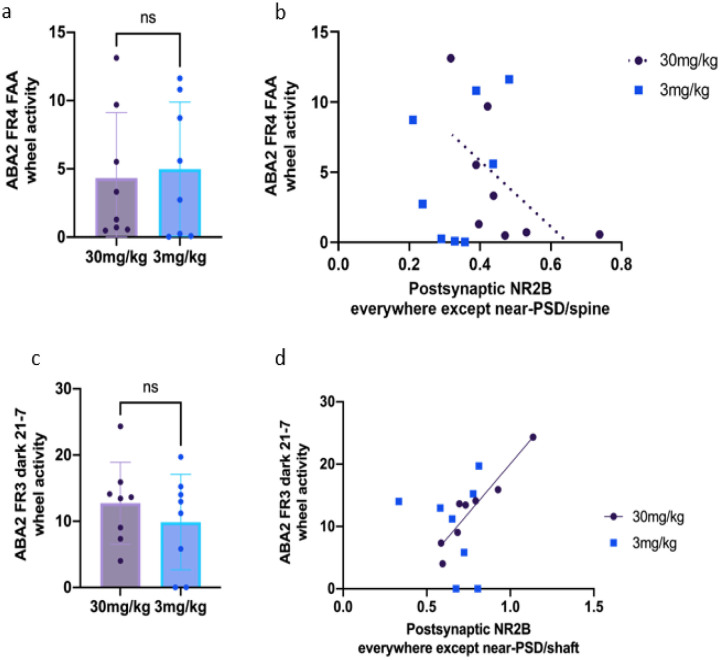
The ameliorative wheel activity effect of the 30 mg/kg ketamine during ABA2 was associated with decreased NR2B at excitatory synapses on GABA-IN **Panels a and c:** There was no significant difference between wheel activities of the two cohorts on ABA2 FR4 FAA or ABA2 FR3 dark 21 –7. **Panel b:** At excitatory synapses of pyramidal neurons’ dendritic spines, there was a negative trend between wheel activity during FAA of ABA2 FR4 and the proportion of postsynaptic NR2B at all locations except near PSD for the 30mg/kg cohort and no correlation for the 3mg/kg cohort. **Panel d:** At excitatory synapses of GABA-IN, there was a significant positive correlation between wheel activity during the postprandial dark hours (9 pm to 7 am) on ABA2 FR3 of the 30 mg/kg cohort and the proportion of postsynaptic NR2B at all locations except near PSD. No such correlation was found for the 3 mg/kg cohort.

Figures 8a and 8b show the day-by-day values of R-values of Pearson correlation between wheel running during different time zones with NR2B immunoreactivity. Similar to the pattern observed for food consumption, there were clear differences in the correlation patterns between the two cohorts with regard to wheel activity. Note that wheels were absent during recovery phases, thereby precluding correlation analyses during the recovery days.The 30 mg/kg cohort exhibited similar dynamics of R-values across the different NR2B locations. During FAA, the 30 mg/kg cohort, but not the 3mg/kg cohort, exhibited a dip of R-values for most NR2B locations except for the location at-PSD (Fig. 8b). This difference across the cohorts prompted us to look more closely at the correlations between wheel running and NR2B labeling during FAA (Fig. 9).

During the ABA2 FR period, both doses yielded significant reductions in averaged wheel running per day compared to the vehicle cohort (Chen et al. 2018). Although wheel activity between the two ketamine cohorts were not significantly different (mean ± SEM in units of km: 6.106 ± 1.69 for the 30 mg/kg cohort; 1.656 ± 1.74 for the 3 mg/kg cohort, p = 0.7938 by the unpaired t-test, Fig. 9a), correlation analysis revealed differences across the cohorts. Specifically, at excitatory synapses of dendritic spines of pyramidal neurons of the 30 mg/kg cohort, a negative trend was found between wheel activity and the proportion of spines labeled for postsynaptic NR2B at all postsynaptic locations except for near-PSD (Pearson’s R=−0.6274, p = 0.0959 for the 30 mg/kg cohort, Fig. 9b), indicating that those individuals with minimal FAA were those that had reduced NR2B expression at excitatory synapses of pyramidal neurons the most. No such trend was found in the 3 mg/kg cohort (Pearson’s R = 0.3822, p = 0.3500 for the 3 mg/kg cohort, Fig. 9b).

#### Correlations at axo-shaft excitatory synapses of GABA-IN.

Axo-shaft excitatory synapses of the 30 mg/kg cohort exhibited higher correlations between NR2B and wheel running than at axo-spinous synapses, especially during the dark hours of FR3 (21:00–7:00) (Fig. 8d, 9b). There was no significant group difference in the wheel activity between the two ketamine cohorts on ABA2 FR3’s dark hours of 21:00–7:00 (mean ± SEM in units of km: 12.73 ± 2.18 for the 30 mg/kg cohort, 9.87 ± 2.46 for the 3 mg/kg cohort, p = 0.4086 by unpaired t-test, Fig. 9c). Yet, for the 30 mg/kg cohort, there was a highly significant positive correlation between wheel activity on ABA2 FR3 during dark phase 21:00–7:00 and the proportion of dendritic shafts of GABA-IN immunolabeled for postsynaptic NR2B at all postsynaptic locations other than near the PSD (Fig. 9d, Pearson’s R = 0.9437, p = 0.0004 for the 30 mg/kg cohort). No such correlation was found in the 3 mg/kg cohort (Fig. 9d, Pearson’s R = 0.1652, p = 0.6959 for the 3 mg/kg cohort).

Data reported were also analyzed by correlating wheel running and the levels of dendritic spines or shafts immunolabeled for NR2B. Correlation analyses counting every single NR2B immunolabel and normalizing the sum by the number of spines or shafts also revealed significance for the same sets of data.

Together, these data indicate that the dose of 30 mg/kg was more efficacious in promoting ABA resilience measured as reduced wheel running through gains of NR2B at axo-spinous excitatory synapses of pyramidal neurons and decreased NR2B-immunoreactivity at axo-shaft excitatory synapses of GABA-IN. These observations are the opposite of the previous finding pertaining to the mPFC of animals without ketamine treatment (Santiago et al. 2021). The current data suggest that ketamine may mediate plasticity of the mPFC synaptic circuitry by unmasking mPFC pathways supporting adaptive behaviors, such as to *suppressthe* hunger-evoked hyperactivity. Moreover, ketamine promoted synaptic changes in opposite directions depending on the cell type (pyramidal neuron vs GABA-IN), as was described above for the ketamine-induced changes supporting food consumption.

### Correlations Between Body Weights and the Proportions of Excitatory Synapses Immunolabeled for NR2B

**Figure 10:**
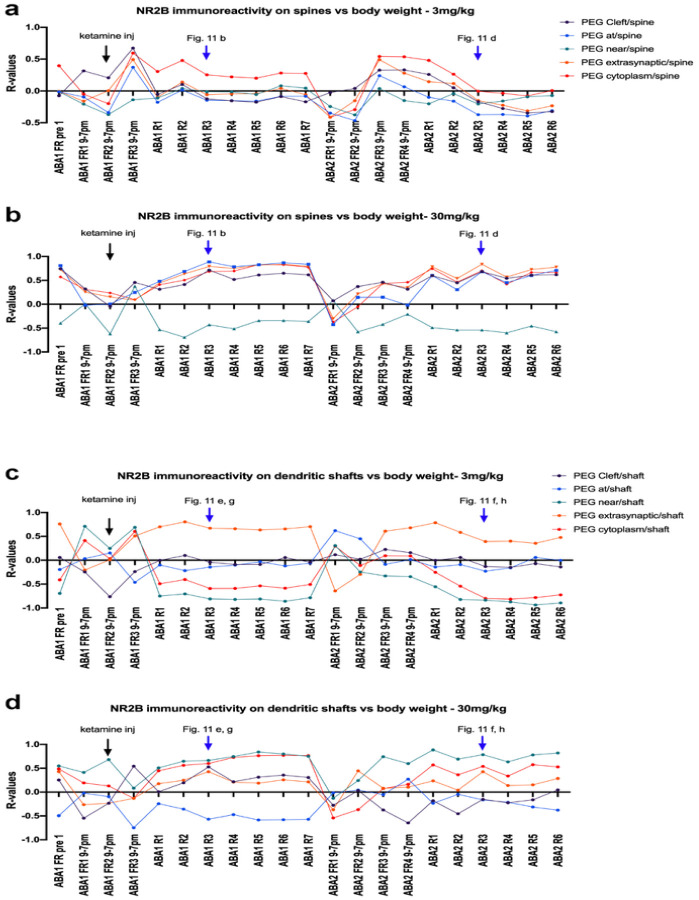
R-values of Pearson correlation analyses throughout the course of ABA1 and ABA2 between body weight and proportions of NR2B at excitatory synapses **Panels a and b:** Axo-spinous synapses on pyramidal neurons. 3mg/kg cohort’s R values shown in panel a, 30mg/kg cohort’s shown in panel b. **Panels c and d:** Axo-shaft synapses on GABA-IN. 3mg/kg cohort’s R-values shown in panel c, 30mg/kg cohort’s R-values shown in panel d.

**Figure 11:**
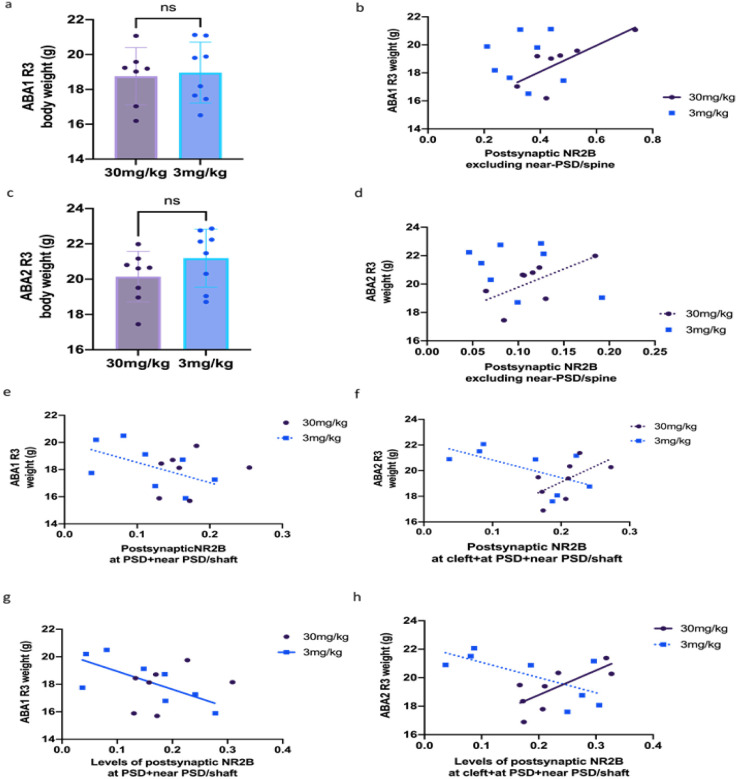
Improvement of body weight retention among the 30 mg/kg cohort was contributed by increased NR2B at excitatory synapses on pyramidal neurons, together with decreased NR2B at excitatory synapses on GABA-IN during recovery from ABA1 and by the increased NR2B at excitatory synapses on GABA-IN during recovery from ABA2. **Panels a and b:** On both ABA1 R3 and ABA2 R3, there was no significant body weight difference, between the two cohorts. **Panels c and d:** Analysis of excitatory synapses on dendritic spines of pyramidal neurons revealed a positive correlation between body weight and the proportion of postsynaptic NR2B at all locations except near PSD of dendritic spines for the 30mg/kg cohort on ABA1 R3, which weakened by ABA2 R3. No correlation was detected for the 3 mg/kg cohort., **Panel e:** Analysis of excitatory synapses on dendritic shaft of GABA-IN revealed a negative trend between body weight and the proportion of postsynaptic NR2B at PSD and near PSD of the 3 mg/kg cohort but not for the 30 mg/kg cohort on ABA1 R3. F: There was a negative trend between body weight and the proportion of postsynaptic NR2B at cleft, at PSD, and near PSD of excitatory synapses of GABA-IN of the 3mg/kg cohort on ABA1 R3 and a positive trend for the 30mg/kg cohort on that day. **Panels g and h:** The levels of NR2B, instead of the proportions of excitatory synapses with NR2B was used in these correlation analyses. A significantly negative correlation was found between NR2B at and near PSD of GABA-IN and body weight of the 3 mg/kg cohort on ABA1 R3. A significantly positive correlation was also found between NR2B at the cleft, PSD and near PSD of GABA-IN of the 30 mg/kg cohort on ABA2 R3.

Body weight would be expected to be affected jointly by food consumption and wheel activity, each of which are regulated by specific synaptic circuits within mPFC (Santiago et al. 2021; Du et al. 2022). Pearson’s correlation analyses revealed correlations between body weight and the levels of postsynaptic NR2B at excitatory synapses of dendritic spines, presumably of pyramidal neurons and dendritic shafts, presumably of GABA-IN. Similar to correlations between NR2B immunoreactivity and food consumption (Fig. 6), correlation patterns of the two cohorts also differed for body weights (Fig. 10). R-values of the 3 mg/kg cohort were low during the two recovery phases and higher during FR periods. Also, the R-value dynamics of postsynaptic NR2B of the 3 mg/kg cohort were uniform across all synaptic locations (Fig. 10a). Oppositely, the 30 mg/kg cohort exhibited high R-values during the two recovery phases and lower R-values during FR periods. Within the 30 mg/kg cohort, postsynaptic NR2B near the PSD had a different R-value dynamic than NR2B at all other locations, as those near the PSD exhibited negative R-values during most of the course of ABA (Fig. 10b). Overall, ABA1 R3 and ABA2 R3 stood out as the days showing the strongest correlation between body weight and NR2B at excitatory synapses of pyramidal neurons’ spines and GABA-IN’s dendritic shafts. These correlations are shown in greater detail in Fig. 11.

#### Correlations at axo-spinous excitatory synapses on pyramidal neurons.

Both the 3 mg/kg and 30 mg/kg cohorts responded to the single ketamine injection with increased body weight, relative to the vehicle group at recovery following ABA2, and only the 30mg/kg cohort improved in weight gain at recovery following ABA1 and during ABA2 FR period (Chen et al. 2018). However, two sample mean t-tests showed no differences between the body weights of the 3 mg/kg and 30 mg/kg ketamine cohorts on any of the days of recovery, including ABA1 R3 or ABA2 R3 (mean ± SEM in units of grams: 17.8 ± 0.619 for 30 mg/kg cohort, 18.3 ± 0.618 g for the 3 mg/kg cohort, p = 0.8199 on ABA1 R3, Fig. 11a; mean ± SEM in units of grams: 19.2 ± 0.507 for the 30 mg/kg cohort; 20.12 ± 0.580 for the 3 mg/kg cohort, p > 0.05 for ABA2 R3, Fig. 11c). In spite of the lack of difference in body weights between the two cohorts, comparisons of the correlations revealed differences across the doses. On ABA1 R3, a significant positive correlation was found between body weights and the proportion of spines immunolabeled for all postsynaptic NR2B other than near-PSD of the 30 mg/kg cohort but not the 3 mg/kg cohort (Pearson’s R = 0.7667, p = 0.0443 for the 30 mg/kg cohort and Pearson’s R = 0.0048, p = 0.9911 for the 3 mg/kg cohort, Fig. 11b). Similarly, on ABA2 R3, a positive correlation trend was found for the 30 mg/kg cohort but not the 3 mg/kg cohort (Pearson’s R = 0.6316, p = 0.0930 for the 30 mg/kg cohort and Pearson’s R = 0.3655, p = 0.3732, Fig. 11d). A positive correlation for the 30 mg/kg cohort (Fig. 11d) was still present when body weight was averaged from ABA1 R2-R7 (not shown), suggesting ultrastructural redistribution of NR2B that occurred and stabilized shortly after the end of ABAI’s FR period.

Correlation analyses for the same days and ultrastructural location of NR2B revealed equally significant outcomes using NR2B levels at excitatory synapses instead of the proportions of NR2B-immunoreactive excitatory synapses.

#### Correlations at axo-shaft excitatory synapses on GABA-IN.

On ABA1 R3, a negative trend was found for the correlation between body weight and the sum of the proportion of shafts immunolabeled for postsynaptic NR2B at and near the PSD of excitatory synapses of GABA-IN of the 3 mg/kg cohort. No correlation or trend was found for the 30 mg/kg cohort (Pearson’s R = 0.2033, p = 0.6620 for the 30 mg/kg cohort and Pearson’s R=−0.5531, p = 0.1551 for the 3 mg/kg cohort, Fig. 11e). On ABA2 R3, there was also a negative trend for the 3 mg/kg cohort between body weight and the proportion of dendritic shafts immunolabeled NR2B at the cleft, at PSD, and near PSD of GABA-IN (Pearson’s R=−0.5995, p = 0.1162 for the 3 mg/kg cohort, Fig. 11f). Oppositely, a positive trend was found in the 30 mg/kg cohort (Pearson’s R = 0.5946, p = 0.1200 for the 30 mg/kg cohort, Fig. 11f).

The same behavioral data set was correlated to NR2B levels, assessed by counting every single NR2B immunolabel and normalizing the sum by the number of dendritic shafts encountered. The outcome revealed greater significances of correlations (Fig. 11g, h). In contrast to the negative trend shown in Fig. 11e for the proportions of synapses immunolabeled, there was a significantly negative correlation between body weight and the level of dendritic shafts immunolabeled for NR2B for the 3mg/kg cohort on ABA1 R3, and no correlation or trend for the 30mg/kg cohort (Pearson’s R = 0.3562, p = 0.4330 for the 30mg/kg cohort and Pearson’s R=−0.7176, p = 0.0450 for the 3mg/kg cohort, Fig. 11g). Likewise, in contrast to the negative trend shown in Fig. 11f, there was a significant positive correlation between body weight and the level of dendritic shafts immunolabeled for NR2B for the 30mg/kg cohort but only a negative trend for the 3mg/kg cohort (Pearson’s R = 0.7428, p = 0.0348 for the 30mg/kg cohort and Pearson’s R=-0.6738, p = 0.0670 for the 3mg/kg cohort, Fig. 11h).

Overall, at dendritic spines of pyramidal neurons, the increase in the proportion and levels of NR2B had an ameliorative effect on body weight restoration and more so for the 30 mg/kg dose than for the 3 mg/kg dose (Fig. 11b). In sharp contrast to the changes at dendritic spines, the changes at excitatory synapses on GABA-IN reflected losses of NR2B of 3 mg/kg cohort, seen as ameliorative for weight retention, changing to a positive correlation by ABA2 and enhanced NR2B that contributed to 30 mg/kg cohort’s body weight gain.

## Discussion

Ketamine is an open channel NMDAR blocker. As such, ketamine that was injected while animals exhibited food restriction-evoked maladaptive hyperactivity and suppressed food consumption may have blocked excitatory synapses within circuits that were mediating those maladaptive behaviors. We hypothesize that ketamine’s blockade of those synapses within mPFC circuitry subserving maladaptive behaviors may have enabled the competing adaptive behaviors’ synaptic circuits to become more dominant, thereby enabling a switch of animals’ behavior from the maladaptive to more adaptive ones. In the following sections, we consider the types of mPFC circuitry and directions of NR2B changes at synapses that would support this hypothesis.

### Food Consumption During Recovery Following Ketamine Treatment

#### The contribution by pyramidal neurons’ postsynaptic NR2B in enhancing food consumption

**Figure 12:**
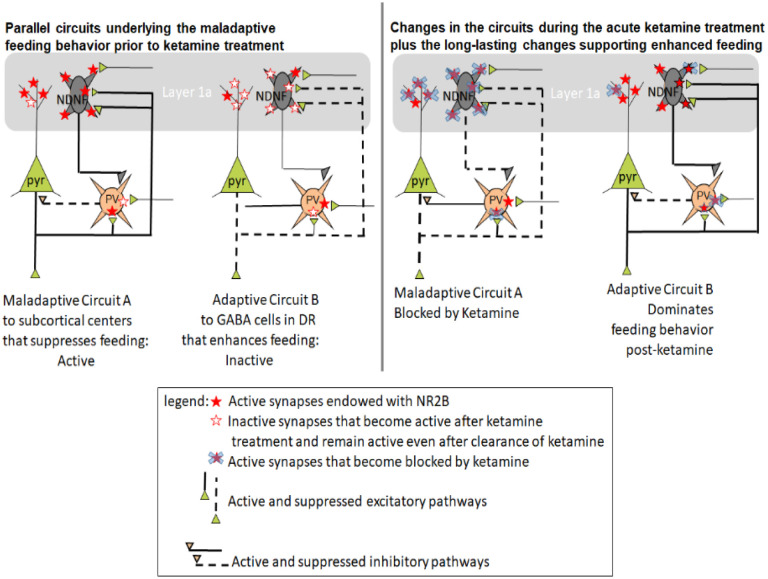
NR2B activity at synapses of parallel circuits within the mPFC regulating food consumption With the efficacious dose of ketamine (30 mg/kg), blockade of the more active synapses in Circuit A (left panel prior to ketamine changing to the circuit in the right panel after ketamine) leads to the emergence of the more adaptive behavior mediated through Circuit B (left panel prior to ketamine, right panel after ketamine). Circuit B’s activity is also enhanced due to increased expression of NR2B at Circuit B’s pyramidal neurons and on GABA-IN (see Fig 7f), many of which are known to express NDNF in layer 1 a and excite pyramidal neurons (pyr) through disinhibition of parvalbumin+ GABA-IN (PV).

Behaviorally, we observed that 30 mg/kg ketamine protected mice against ABA vulnerability by increasing food consumption during ABA and during the recovery phase following both ABA1 and ABA2. During recovery from ABA1, there was a positive trend for the 30 mg/kg dose and not for the 3 mg/kg dose between increased food consumption and NR2B immunoreactivity at excitatory synapses on pyramidal neurons. This trend suggests that the more efficacious dose of 30 mg/kg following ABA1 may have been due, in part, to an increase in the proportion of dendritic spines of pyramidal neurons immunolabeled for NR2B. The positive correlation was observed almost everywhere (at PSD, extrasynaptically, in the synaptic cleft, and cytoplasmically) (Fig. 7b). This fits with a previous finding indicating that an increase of the E/I (excitatiion to inhibition) ratio of synaptic inputs to mPFC pyramidal neurons which are excitatory themselves and project to multiple subcortical regions promotes food consumption (Du et al. 2022). One known target of the mPFC pyramidal neurons promoting food consumption is the GABA-INs in dorsal raphe (mPFC→DR_GABA_) (Jankowski et al., 2003), presumably by inhibiting the firing of serotonergic neurons in DR (Nectow et al. 2017). Our new finding suggests that 30 mg/kg of ketamine may have been more efficacious than the 3 mg/kg dose of ketamine for increasing the E/I ratio of this pathway within mPFC and/or enhancing the strength of mPFC→DR afferents forming synapses onto GABA-IN in DR (Fig. 12, Circuit B after Ketamine).

Following ABA2, the efficacious dose of 30 mg/kg correlated not at all with NR2B levels at excitatory synapses onto pyramidal neurons in mPFC (Fig. 7d). This could be due to the disengagement of the mPFC→DR_gaba_ pathway as the major contributor to the enhanced food consumption. More likely, the improved food consumption is due to a counterbalanced weakening of another mPFC excitatory outflow that suppresses food consumption (Circuit A after Ketamine in Fig. 12), such as the mPFC pyramidal neurons that directly target serotonergic neurons in DR (mPFC◊DR_5HT_) (Nectow et al. 2017; Jankowski and Sesack 2004). In the PFC, there are multiple populations of pyramidal neurons that regulate food consumption. Besides those projecting to DR, mPFC neurons that project to lateral hypothalamus (LH) promote cued food consumption of non-starving animals (Holland and Petrovich 2005) but can also suppress feeding even of food-deprived animals, if it targets the glutamatergic neurons in LH (mPFC→LH_glut_) (Jennings et al. 2013). Another excitatory outflow from the mPFC to the lateral habenula (mPFC→LHb) is theorized to underlie stress-evoked anxiety and anhedonia in humans (Vadovicova 2014). The excitatory outflow from mPFC suppressing either of these food consumption-suppressing subcortical centers may have been weakened by the 30 mg/kg dose of ketamine in time for recovery from ABA2, thereby diminishing the positive correlation trend seen after ABA1. Published work already exists indicating that the prophylactic stress-buffering effects of ketamine involve synaptic plasticity of the mPFC→DR that targets the 5-HT neurons in DR (Dolzani et al. 2018).

The idea that there are parallel antagonistic pathways from mPFC for food consumption regulation is supported by considering the outcome of treating ABA animals with the inefficacious dose of 3 mg/kg. The outcome was that food consumption correlated negatively and strongly with NR2B prevalence at excitatory synapses formed on pyramidal neurons. This may reflect the persistent dominant influence by the food consumption-suppressive excitatory outflow from mPFC (mPFC→LH_glut_. mPFC→LHb; mPFC◊DR_5HT_) described above (Circuit A in Fig. 12), when the synaptic circuitry underlying the maladaptive behavior is not blocked sufficiently by ketamine. Further studies of the synaptic circuitry within DR and mPFC would be necessary to understand more fully about the mechanism of action of the efficacious dose of ketamine.

#### The contribution of GABA-IN’s postsynaptic NR2B in the enhanced food consumption.

Our earlier study that did not employ ketamine treatment showed that chemogenetic stimulation of the mPFC→DR pathway (Circuit B in Fig. 12) could not override the strong GABAergic inhibition within mPFC that inhibited feeding (Du et al. 2022). The efficacious dose of ketamine may have blocked the active synapses underlying maladaptive behavior (Circuit A after Ketamine in Fig. 12). This ketamine-mediated blockade of the excitatory synapses on GABA-IN in Layer 5 (Circuit A after Ketamine, Fig. 12) may have enabled Circuit B’s excitatory outflow from the mPFC to dominate behavior as ketamine’s acute mechanism in addition to the changes hypothesized above regarding NR2B expression at excitatory synapses on GABA-IN in Layer 1 to support the adaptive changes that began during recovery from ABA1 (Fig. 7e) and lasted for more than 20 days (see Fig. 7f).

In the absence of ketamine, the relative prevalence of GABAergic innervation of Layer 5 pyramidal neurons forming the mPFC→DR pathway correlated negatively with food consumption (Du et al. 2022). Following the treatment with 30 mg/kg ketamine, the correlation between GABA inhibition and food consumption reversed, since higher levels of NR2B expression on GABA-IN in Layer 1 correlated with enhanced food consumption. GABA-IN located in Layer 1, and especially those in layer 1a which was the central focus of our analyses, are reported to be NDNF+ (neuron-derived neurotrophic factor-expressing) GABA-IN, also referred to as neurogliaform cells, that receive excitatory afferents from ventromedial thalamus and inhibit PV + GABA-IN, thereby disinhibiting the pyramidal neurons that are targeted by PV + GABA-IN (Anastasiades and Carter 2021; Anastasiades et al. 2021; Karnani et al. 2016) (Circuits A and B). The increased disinhibition by the NDNF + GABA-IN of the mPFC◊DR pathway, through increased expression of NR2B in these cells is likely to have been a strong contributor to the ketamine-mediated long-lasting enhancement of food consumption (dashed line from PV cells to pyramidal cell bodies of Circuit B after Ketamine). Conversely, the maladaptive behavior exhibited by animals prior to the ketamine treatment may have been contributed by the exuberance of excitatory synapses onto NDNF + GABA-IN in Layer 1 targeting PV + GABA-IN that caused disinhibition of the pathway (dashed lines from PV cells to pyramidal neurons in Circuit A, left panel). Following treatment with 30 mg/kg ketamine, these active synapses would be expected to have become blocked (based on ketamine’s selectivity to block active synapses, see the red stars covered by blue x marks in Fig. 12), thereby reducing the disinhibition of the previously maladaptive pathway and concomitantly strengthened inhibition by PV cells in Circuit A after Ketamine, right panel). It is likely that the positive correlation between NR2B on GABA-IN and food consumption emerged only because of the ketamine-blockade of the previously active maladaptive synapses, thereby enabling the NR2B on NDNF + GABA-IN subserving the adaptive pathway (Circuit B after Ketamine, right panel) to dominate behavior.

### Wheel Running During ABA2,14 days After Ketamine Injection

#### Primary contribution by GABA-IN in the ketamine-mediated suppression of wheel running.

Both the 30 mg/kg and 3 mg/kg ketamine cohorts exhibited significantly less wheel activity during ABA2, compared to the vehicle cohort (Chen et al. 2018). EM analysis pointed to excitatory synapses on GABA-IN as a critical site to support ketamine’s ameliorative actions of suppressed wheel running (Fig. 9d).

**Figure 13:**
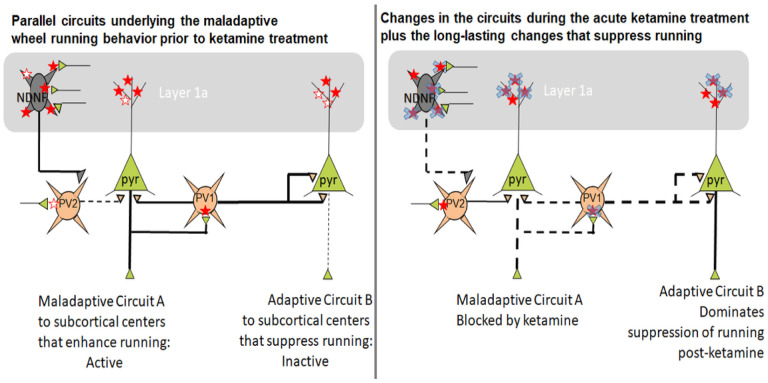
NR2B activity at synapses of parallel circuits within the mPFC regulating food restriction-evoked wheel running **Panel a:** Circuits prior ketamine treatment **Panel b:** Circuits following ketamine injection. With the efficacious dose of ketamine (30 mg/kg), blockade of the more active synapses in Circuit A that mediate maladaptive behavior leads to the emergence of the more adaptive behavior mediated by Circuit B. Circuit As activity is also suppressed due to decreased expression of NR2B at GABA-IN in Layer 1 (Fig 9d). PV1 is recruited by pyramidal neurons (pyr) mediating the maladaptive circuit A, while PV2 is not. See Fig 12 for additional figure legends.

We had learned in an earlier chemogenetic interrogation experiment that mPFC contains at least two parallel antagonistic pathways regulating food restriction-evoked hyperactivity of ABA animals: those that promote wheel running, which project to dorsal medial striatum (mPFC→DMS) and those that project elsewhere and suppress wheel running. Furthermore, we had learned that GABA-IN of the mPFC that are recruited by the chemogenetic stimulation of mPFC→DMS pyramidal neurons mostly innervate cell bodies of those pyramidal neurons that suppress running (Circuit B in Fig. 13) and *not* the cell bodies of pyramidal neurons projecting to DMS (Circuit A in Fig. 13) (Santiago et al. 2021). A change in circuit consistent with the gain of resilience after ketamine treatment is that 30 mg/kg of ketamine blocks the active synapses, and most critically those excitatory synapses on PV + neurons inhibiting the adaptive circuit, enabling the activity of the pyramidal neurons supporting the suppression of running to dominate behavior (Circuit B after Ketamine).

During ABA2, the most prominent correlation of NR2B levels to wheel activity appeared at Layer 1 a excitatory synapses on GABA-IN of the 30 mg/kg cohort. As stated above when discussing the circuits underlying increases in food consumption, the most prevalent type of GABA-IN in Layer 1 a are the NDNF + GABA-IN that mediate disinhibition of pyramidal neurons through inhibition of PV + GABA-IN. Here, the correlation was strongly positive (Fig. 9d), indicating that those individuals with the weakest NDNF + GABAergic inhibition, due to weakened excitatory synaptic input, *minimized* the level of wheel running the most, i.e., gained ABA resilience. This correlation is consistent with the idea that 30 mg/kg of ketamine blocked, then eliminated NR2B expression at excitatory synapses on NDNF + GABA-IN, thereby reducing the disinhibition of the maladaptive Circuit A (Circuit A after Ketamine in Fig. 13). The strong correlation indicates that this reduction of disinhibition in Layer 1 a upon the maladaptive circuit was most influential for reducing the food restriction-evoked hyperactivity. While the NDNF + GABA-IN in Layer 1 a could be recruited following activation of Circuit A pyramidal neurons, it is likely that the PV + GABA-IN targeted by NDNF + GABA-IN are distinct from the population of PV + GABA-IN recruited through activity of Circuit As pyramidal neurons (PV1 cells of Fig. 13, left panel). This is because the PV + cells inhibited by NDNF+ (PV2 cells in the left panel) would have to be those that remained relatively inactive while animals exhibited maladaptive behaviors driven by Circuit A, thereby escaping ketamine’s activity-dependent blockade and becoming active after the ketamine treatment (PV2 cells, in the right panel).

#### Pyramidal neurons’ secondary contribution to the ketamine-mediated suppression of wheel running.

The 30 mg/kg cohort also exhibited a weak negative trend between excitatory synaptic drive of pyramidal neurons and wheel activity (Fig. 9b). This is consistent with the idea that the excitatory synaptic drive of pyramidal neurons that suppress running was enhanced through redistribution of NR2B-NMDARs towards excitatory synapses on pyramidal neurons of the adaptive circuit that had been dormant prior to ketamine treatment (Circuit B after Ketamine, Fig. 13), while excitatory synapses on pyramidal neurons of the maladaptive circuit that had been active during ABA1 became blocked by ketamine (Circuit A after Ketamine), culminating in the slight reduction of NR2B at pyramidal neurons, overall (5 filled stars associated with pyramidal neurons in the left panel to 3 filled starts in right panel).

### Body Weight After Ketamine Injection

#### Contribution by postsynaptic NR2B of pyramidal neurons and GABA-IN in body weight restoration associated with the 30 mg/kg cohort

Body weight is regulated jointly by wheel running and food consumption. However, with regard to ABA1, we observed a significant ameliorative effect of the 30 mg/kg dose of ketamine on body weight restoration during recovery, when the running wheel was no longer accessible to mice (Chen et al. 2018), suggesting that the pathway underlying body weight restoration could have been related to those pathways regulating food consumption more than of wheel running. The prominently positive correlation observed between NR2B levels at pyramidal neurons’ excitatory synapses and body weight restoration following ABA1 (Fig. 11b) suggests that this was contributed largely by the enhanced excitability of the mPFC→DR_gaba_ pathway (Du et al. 2022; Nectow et al. 2017) (Adaptive Circuit B, right panel in Fig. 12). The loss of correlation of NR2B levels at excitatory synapses on GABA-IN for the 30 mg/kg cohort, together with the persistently negative correlations for the cohort that received the ineffective 3 mg/kg dose (Fig. 11e, f, g, h) indicates that the relative difficulty with body weight restoration of the 3 mg/kg ketamine cohort was due to the persistently strong activity of excitatory synapses on Layer 1 GABA-IN that disinhibited the maladaptive behavior (circuit A, left panel), which could be blocked effectively by 30 mg/kg of ketamine (Fig. 12, right).

During ABA2 and recovery from it, the positive correlation between NR2B levels at excitatory synapses on pyramidal neurons and body weight weakened slightly (Fig. 11d), relative to the recovery from ABA1 (Fig. 11b). This suggests that the ameliorative behavior leading to better body weight restoration by the 30 mg/kg dose following ABA1, compared to the 3 mg/kg dose (Chen et al. 2018) is likely to reflect the strong positive correlation of NR2B levels at pyramidal neurons of the adaptive pathway (mPFC→DR_GABA_, Circuit B in Fig. 12) which could be revealed due to ketamine blockade, then removal of NR2B from pyramidal neurons of the maladaptive pathway, leading to weakening of the maladaptive pathway (mPFC→LH_glut_, mPFC→LHb; mPFC◊DR_5HT_) (Circuit A after Ketamine in Fig. 12).

Added to the plasticity at pyramidal neurons is the strengthened suppression of the maladaptive pathway mediated by GABA-IN, reflected by the significantly positive correlation between NR2B levels at excitatory synapses of GABA-IN and better body weight restoration (Figs. 11h). As hypothesized above for the action of the effective dose of ketamine for food consumption, we believe the effective dose of ketamine successfully blocked the maladaptive food consumption pathways, such as the excitatory afferents to Layer 1 GABA-IN that disinhibited the maladaptive pathways (circuit A after Ketamine, Fig. 12), thereby enabling up-regulation of NR2B levels at excitatory synaptic sites on GABA-IN that disinhibit adaptive pathways (Circuit B after Ketamine, Fig. 12), such as the mPFC◊DR_GABA_.

## Conclusion

The site of action of ketamine is likely to be distributed across multiple brain regions, although the literature pertaining to ketamine’s mode of action is dominated by research examining rather selective brain regions - mPFC, hippocampus and LHb. To our knowledge, this is one of only two studies examining ketamine’s effect on synaptic properties as it relates to ABA, an animal model of AN.

Our study is also unique in having examined the impact of ketamine administered during mid-adolescence, while most other studies examined the impact of ketamine upon psychiatric diseases and animal models in adulthood. Adolescence is when multiple mental illnesses arise (Whitford et al. 2007), and anorexia nervosa (AN) is one such mental illness that emerges most prominently during adolescence (Aoki and Santiago 2022). This is the reason we chose mid-adolescent female mice to be the subjects of our study. Brain maturation and development, including pruning, are very active during adolescence in mPFC, especially in response to stress (Whitford et al. 2007; Juraska and Willing 2017; Anderson 2022; Delevich et al. 2021). The active turnover of synapses during mid-adolescence may have enhanced the receptivity of mPFC to ketamine more than in adulthood, since administration of the same ketamine treatment to animals undergoing ABA during late adolescence/early adulthood was not efficacious (Aoki 2020). We are currently exploring alternative efficacious doses of ketamine for ameliorating ABA vulnerability in adulthood.

Our overall finding of this initial study is that the correlations between NR2B-NMDAR levels at synapses in layer 1 a of mPFC and variables related to ABA vulnerability (wheel activity, food consumption, body restoration) require the consideration of plasticity at multiple excitatory corticofugal pathways that work antagonistically. For example, we had learned from earlier chemogenetic studies (Santiago et al. 2021) that there must be two or more pathways of mPFC origin subserving enhancement versus suppression of food restriction-evoked hyperactivity. The current findings enabled us to recognize that the effective dose of ketamine ameliorated ABA vulnerability by suppressing certain pathways while boosting others. By incorporating the knowledge from prior studies that interrogated the mPFC excitatory outflows chemogenetically, we could surmise that the suppressed pathways might include the mPFC◊DMS (Fig. 13) but are likely to include additional parallel pathways that suppress food intake (Fig. 12). Conversely, we hypothesize that pathways enhanced in synaptic strength through increased levels of NR2B might include the mPFC◊DR_GABA_ pathway (Fig. 12) but is also likely to include mPFC corticofugal pathways that suppress wheel running (Fig. 13). Moreover, we learned that NR2B expression at excitatory synapses onto GABA-IN exhibit plasticity that supports resilience most consistently (strong correlations), yielding both reduced wheel running (Fig. 9d and Fig. 13), increased food consumption (Figs. 7e and 7f; Fig. 12) and increased body weight (Figs. 11g and 11h; Fig. 12).

The multi-directional and multiple pathways that are differentially modulated across the efficacious versus inefficacious doses fit with the idea hypothesized at the onset – namely that 30 mg/kg of ketamine, more so than the 3 mg/kg dose, blocks the active pathways subserving maladaptive behaviors, thereby enabling the relatively quiet pathways to emerge as dominant players supporting adaptive behavior. Future studies that extend the analysis to additional brain regions promise to provide a more complete understanding of the mechanism of action of ketamine. Moreover, analysis of NR2B redistribution evoked by sub-anesthetic doses of ketamine at excitatory synapses of definitively identified corticofugal pathways would greatly facilitate our knowledge of the multiple parallel pathways of the mPFC that coordinate the switching of maladaptive behaviors to adaptive behaviors.

## Data Availability

All data not included in the manuscript can be made available upon request.
